# Technical updates to basic proteins focalization using IPG strips

**DOI:** 10.1186/1477-5956-10-54

**Published:** 2012-09-06

**Authors:** Jordane Dépagne, François Chevalier

**Affiliations:** 1 Proteomic Laboratory, iRCM / DSV / CEA, Fontenay aux Roses, France

**Keywords:** Two-dimensional electrophoresis, Proteomic methods, Isoelectric focusing, IPG, Cup-loading, Paper bridge loading, Basic proteins

## Abstract

**Background:**

Gel-based proteomic is a popular and versatile method of global protein separation and quantification. However, separation of basic protein still represents technical challenges with recurrent problems of resolution and reproducibility.

**Results:**

Three different protocols of protein loading were compared using MCF7 cells proteins. In-gel rehydration, cup-loading and paper-bridge loading were first compared using 6–11 IPG strips, as attempted, in-gel rehydration gave large horizontal steaking; paper-bridge loading displayed an interesting spot resolution, but with a predominant loss of material; cup-loading was selected as the most relevant method, but still needing improvement. Twelve cup-loading protocols were compared with various strip rehydration, and cathodic wick solutions. Destreak appeared as better than DTT for strip rehydration; the use of isopropanol gave no improvement. The best 2DE separation was observed with cathodic wicks filled with rehydration solution complemented with DTT. Paper-bridge loading was finally analyzed using non-limited samples, such as bovine milk. In this case, new spots of basic milk proteins were observed, with or without paper wicks.

**Conclusion:**

According to this technical study of basic protein focalization with IPG strips, the cup-loading protocol clearly displayed the best resolution and reproducibility: strips were first rehydrated with standard solution, then proteins were cup-loaded with destreak reagent, and focalisation was performed with cathodic wicks filled with rehydration solution and DTT. Paper-bridge loading could be as well used, but preferentially with non-limited samples.

## Background

Two-dimensional electrophoresis is until now one of the most widely used technique for performing functional proteomics [1]. Indeed, it has the capacity to separate, visualize and quantify several thousands of proteins in a single gel from a complex biological sample, allowing the large-scale analysis of protein expression differences. Gel-to-gel variability has been largely improved thanks to the use of IPG strips and robust gel staining protocols [2,3].

But even now, IEF in the alkaline region is considered as a challenge to separate basic proteins by 2-DE and most of gel-based proteomic studies were performed in the acidic range. Basic proteins are difficult to separate in the first dimension for several reasons. At pH above 8, polymerized acrylamide gel is unstable, and can degrade. So it is very important to use only fresh strips stored at a correct temperature. A degradation of acrylamide can be visualized when the plastic part of the dry strip glues to the acrylamide gel part, with a tendency to twist.

At basic pH, it is mandatory to keep the redox status for cysteines using reducing agent such as DTT [4]. But a problem concerns the migration of DTT towards the anode during IEF at basic pH. This results in depletion of DTT at the cathode and leads to reformation of intra- and inter-molecular disulphide bridges due to the oxidation of sulphydryl groups. DTT is a weak acid and hence it is transported out of the basic part of the IPG strip during focusing, resulting in horizontal streaking [5]. This can cause proteins within the sample to become less soluble, leading to horizontal streaking within the gels and thus poor resolution of the basic proteins. Different sample application methods were alternatively proposed to overcome these problems, such as anodic cup-loading, in-gel rehydration, or paper-bridge loading. The addition at the cathodic electrode of a paper wick soaked with DTT was as well proposed to replenish IPG strip with new DTT during focalisation. The use of destreak reagent was proposed to replace DTT to limit unspecific oxidation of protein thiol groups, a combination of isopropanol and glycerol in rehydratation buffer was reported to improve the focusing of proteins in the alkaline IPG strips [6,7]. The paper-bridge loading protocol allowed an improvement of resolution for preparative loading of cardiac mitochondrial samples in the 6–11 pI range [8].

Finally, electroendosmotic flow was reported to occur at high pH values, leading to a basic gap characterized by a zone of streaking and poorly focused proteins close to the cathode. The presence of hydroxide ions in the IPG-strip causes electro-osmotic pumping from the cathode towards the anode with the result that water present in the cathodic paper wick is pumped into the IPG-strip. The authors reported that the gap can therefore be avoided by the use of solutions containing urea in the cathodic electrode wick and DTT should be avoided in the wick as the transport of these components into the IPG-strip will intensify the electro-osmotic transport from the paper wick [9].

In the present article, we first evaluated the separation capacities of three conventional protein loading methods using basic IPG strips. Different conditions and samples were then compared to optimize the cup-loading and the paper-bridge loading protocols.

## Results and discussion

### Evaluation of methods for protein loading

**Figure 1:**
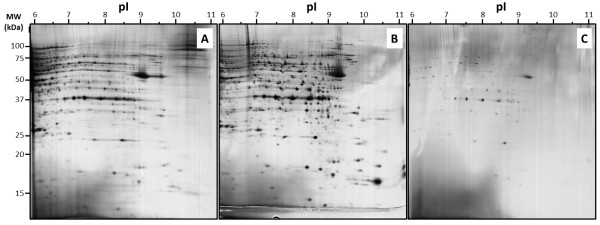
**2DE separation of MCF7 basic protein: iso-electric focalisation was performed with 50 μg proteins using 6–11 pH IPG strip and 12% acrylamide was used for the second dimension.** Proteins were loaded by in-gel rehydration (**A**), anodic cup loading (**B**), and paper-bridge loading (**C**).

To analyse the capacity of IPG strips to separate protein, we first compare three protocols of protein loading, with 50 μg proteins (Figure 1). Almost the same buffer conditions were used to investigate the efficiency of each protocols, the same protein sample and the same protein amount were used, *ie*, 50 μg of MCF7 total extract in TUC solution (7 M urea / 2 M thiourea / 4% CHAPS).

In case of protein loading during strip rehydration (Figure 1A), proteins were mixed with RB (7 M urea, 2 M thiourea, 4% CHAPS, 0.05% triton X100, 5% glycerol) complemented with 1.2% destreak reagent and 0.5% IPG buffer 6–11. Large horizontal smears were observed with most of abundant proteins spots, showing a weak focalisation capacity. This observation was in agreement with previous studies, indeed, protein loading during strip rehydration wasn’t recommended in case of basic IPG strip [5].

In case of protein loading with the cup loading protocol (Figure 1B), strips were rehydrated with RB complemented with 1.2% destreak reagent and 0.5% IPG buffer 6–11. Protein sample was applied after strip rehydration using a plastic cup. Electrode wicks were soaked with 50 μL of water. Lots of spots appeared as well focalized but some horizontal smears were still observed with the most abundant proteins spots.

In case of protein loading with the paper-bridge loading protocol (Figure 1C), strips were rehydrated with the same buffer conditions as the cup loading protocol. Protein sample was applied after strip rehydration onto the anodic electrode wicks, the cathodic electrode wicks was soaked with 50 μL of water. Very few “well-focalized” spots can be distinguished with this last condition. It appeared clearly that almost 90% of proteins were lost.

We decided to focus our interest on the cup loading protocol as it is the most widely used protocol for basic proteins, and as it appeared to be the most efficient loading protocol in our conditions.

### Tests of various buffer conditions with the cup loading protocol

For cup-loading, IPG dry strips are first rehydrated with rehydration buffer containing at least urea, nonionic and / or zwitterionic detergents, a reducing agent and small amounts of carrier ampholytes [5]. Since several decades, protocol developments were proposed to improve basic protein solubilisation and focalisation. DTT was replaced by destreak agent [9], glycerol and isopropanol were included in rehydration buffer [6], but sometimes with contrasted observations, leading to interpretations problems and confusions. Indeed, protein focalisation using IPG strips involved a range of specific and useful chemicals, which interaction could produce unpredictable troubles.

**Figure 2:**
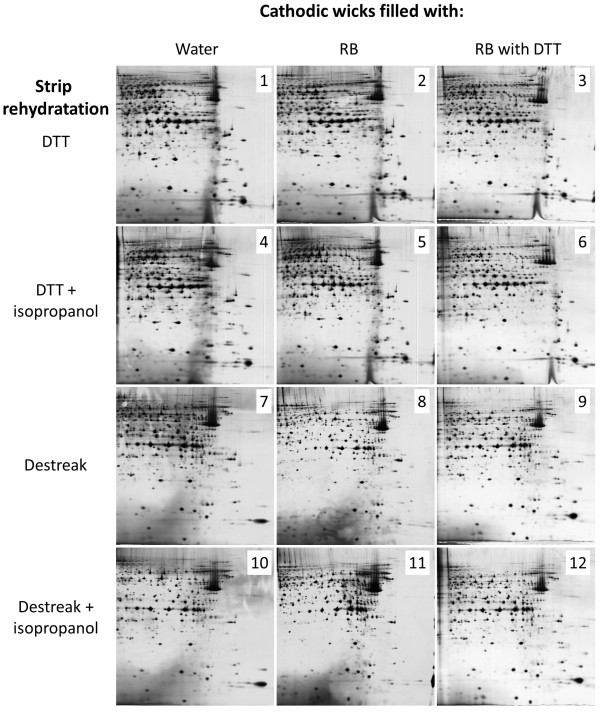
**Comparison of MCF7 basic protein 2DE separation using twelve different anodic cup-loading protocols.** Iso-electric focalisation was performed with 50 μg proteins using 6–11 pH IPG strip and 12% acrylamide was used for the second dimension. The number reported on each picture corresponded to the cup-loading condition used, as presented in detail in Table 1.

An experimental plan was generated to examine DTT, destreak reagent and isopropanol interactions within strip rehydration and the replacement of water by urea with or without DTT onto cathodic wicks (Figure 2). Twelve experimental conditions (methods) were performed and the 2DE maps were compared. An accurate and regular image analysis using the Samespots software appeared quite difficult since many spot groups presented modified focalisation pattern.

Three evident observations could be depicted even without a complete computer assisted analysis: *(i)* firstly, although most of spots were unmodified and reproducible between samples in the left part of pictures (pI 6 to 8.5), major differences were observed in the right / bottom part of 2DE gel pictures; *(ii)* secondly, the use of isopropanol during strip rehydration gave no modification or improvement of spots pattern; *(iii)* finally, spot focalisation was improved when cathodic wicks were filled with RB plus DTT instead of RB without DTT, especially in case of strip rehydration with destreak reagent.

**Figure 3:**
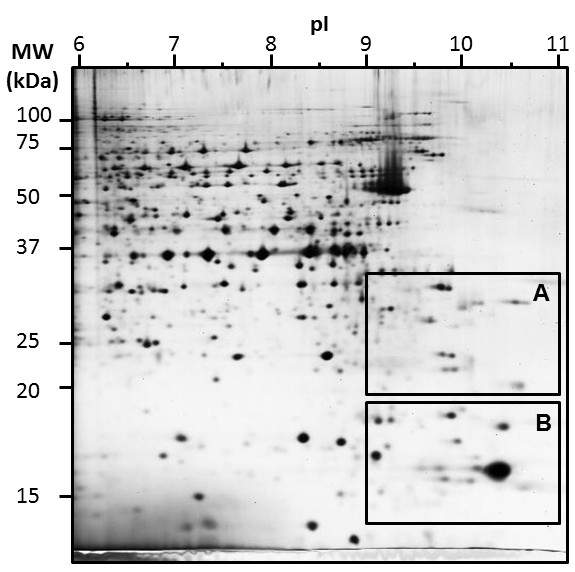
**2DE map of MCF7 basic protein separation using anodic cup-loading protocol n°9, as presented in Figure **2** and Table **1. Specific areas **A** and **B** were further analysed.

**Figure 4:**
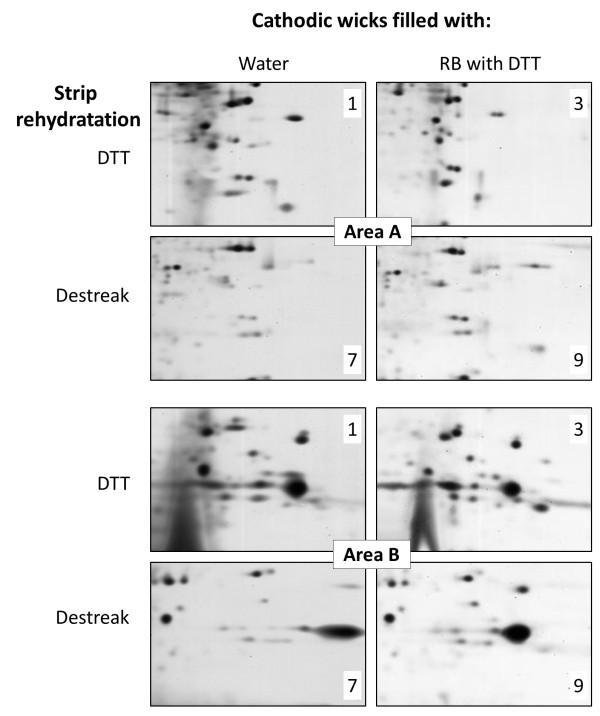
**Picture comparison of areas A and B from Figure **3** as a function of different anodic cup-loading protocols.** The number of spots within each area was estimated by picture analysis and reported in Table 1.

As shown in Figure 3, areas A and B (in the right/bottom part of the 2D gel) corresponded to the 2DE regions of particular interest to evaluate the efficiency of protein spot separation. These specific gel areas were further analysed in detail, using the most different spot patterns. Indeed, from the twelve initial conditions, four were selected and further analysed in detail according to these specific areas (Figure 4 and Table 1): cathodic wicks filled with water or with basic solution complemented with DTT; and strip rehydration using DTT or destreak.

**Table 1:** **Methods tested for basic proteins focalization with the corresponding number of spots in area A and B (Figure** 3**) and an estimation of each focalization efficiency and overall image quality**

**Method**	**Strip rehydration**	**Cathodic wicks**	**Spots area A**	**Spots area B**	**Focalisation efficiency**	**Overall image quality**
**1**	160 mM DTT	50 μl water	82 +/− 9,9	79 +/− 14,1	+/−	--
**2**	160 mM DTT	50 μl RB	73 +/− 7,1	76,5 +/− 3,5	-	-
**3**	160 mM DTT	50 μl RB	67 +/− 4,2	78 +/− 4,2	+/−	+/−
		160 mM DTT				
**4**	160 mM DTT	50 μl water	82 +/−8,5	76,5 +/− 4,9	+/−	--
	10% isopropanol					
**5**	160 mM DTT	50 μl RB	60 +/− 11,3	70,5 +/− 9,2	-	-
	10% isopropanol					
**6**	160 mM DTT	50 μl RB	67,5 +/− 9,2	80,5 +/− 0,7	+/−	+/−
	10% isopropanol	160 mM DTT				
**7**	1,2% destreak reagent	50 μl water	58 +/− 1,4	61,5 +/− 4,9	+/−	+/−
**8**	1,2% destreak reagent	50 μl RB	40 +/− 24	67 +/− 17	+	-
**9**	1,2% destreak reagent	50 μl RB	71,5 +/− 0,7	72,5 +/− 3,5	++	++
		160 mM DTT				
**10**	1,2% destreak reagent	50 μl water	35 +/− 1,4	65,5 +/− 4,9	+/−	+/−
	10% isopropanol					
**11**	1,2% destreak reagent	50 μl RB	36,5 +/− 3,5	59 +/− 5,7	+	-
	10% isopropanol					
**12**	1,2% destreak reagent	50 μl RB	72 +/− 1,4	74 +/− 7,1	++	++
	10% isopropanol	160 mM DTT				

In case of strip rehydration with DTT, the use of cathodic wicks filled with RB plus DTT (method 3) instead of water (method 1) allowed a clearer background but without improvement of spot focalisation (Figure 4). In case of cathodic wicks filled with water, the use of desteak reagent (method 7) instead of DTT (method 1) during strip rehydration allowed a better focalisation, but with a decrease in some spot intensity. It appeared that a lot of spots were common between methods 1 and 9, with a higher number of spots in case of method 1, but with a better spot repeatability and an enhanced overall image quality in case of method 9 (Table 1). The use of destreak reagent for strip rehydration, combined with cathodic wicks filled with RB plus DTT (method 9) appeared as the best compromise to give an acceptable reproducibility and the best focalisation efficiency and overall image quality using the cup-loading protocol.

### Paper-bridge loading protocol investigation

Although the paper-bridge loading protocol resulted in a massive loss of protein, this protocol could be useful in case of study of non-limiting protein samples. Actually, it offers a good separation of proteins with few horizontal smears [8]. We decided to work with bovine milk proteins, a largely available source of proteins, and with major nutritional interests [10,11]. These proteins were studied many times using two-dimensional electrophoresis and proteomic tools to reveal a large complexity in term of post-translational modifications and protein relationship [12-14]. Most of bovine milk proteins were focused in the 4–7 pH range, such as caseins (alpha S1, alpha S2, beta and kappa), beta-lactoglobulin and alpha-lactalbumin [15-17]. A paper-bridge strategy to load milk protein in the basic range could be a way to deplete the sample of the most abundant proteins, showing specifically and with a higher resolution basic proteic component.

**Figure 5:**
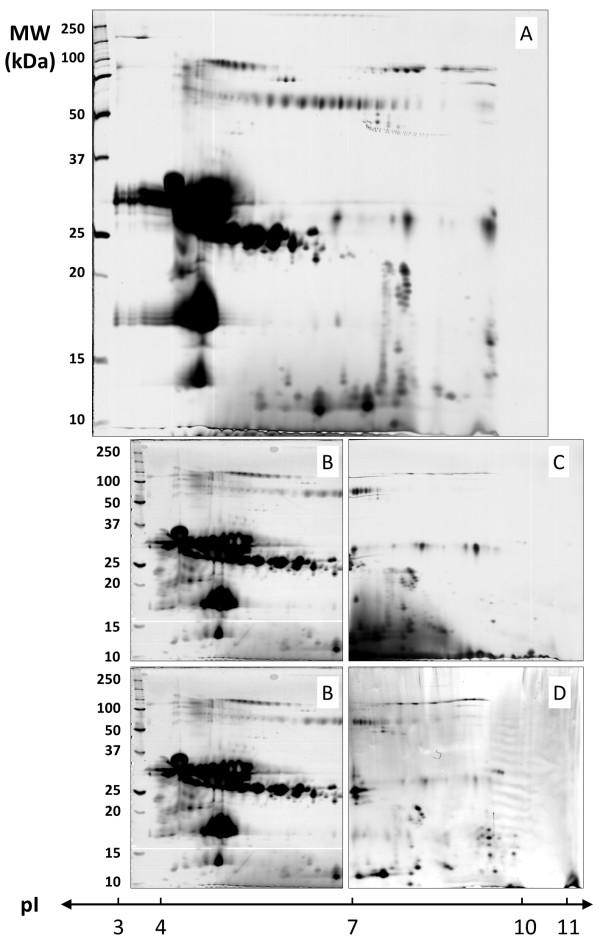
**2DE map comparison and alignment of bovine milk proteins using in-gel rehydration or paper-bridge loading.** 600 μg of non-fat dry milk proteins were in-gel loaded using 3–10 IPG strips (**A**) or paper-bridge loaded using 6–11 IPG strips with (**C**) or without electrode wicks (**D**) during focalization. In a second time, paper-bridge previously used with 6–11 IPG strip was applied with 4–7 IPG strips (**B**) to focalize remaining proteins.

A conventional protein separation was first performed with protein loading during strip rehydration using a 3–10 pH range (Figure 5A). As attempt, and according to previous results, the major milk proteins were observed in the left part of the gel, with only few proteins in the right part.

**Figure 6:**
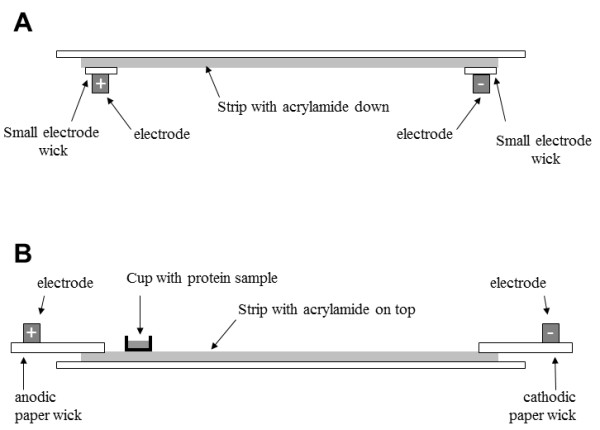
**Schematic of IPG strip, electrodes and paper wicks positions as function of protein loading protocols. A**: sample in-gel rehydration of IPG strip, with acrylamide face down. **B**: cup-loading of protein sample, with acrylamide face up, a plastic sample cup was placed at the anodic end, paper wicks connected strip with electrodes.

The paper-bridge loading protocol was test in a first time with paper wicks placed at both electrodes, and with an anodic application of the milk sample (Figure 6A). Protein sample was loaded from wick into the strip during 16 hours at 200 V according to [8]; anodic wick was then removed and replaced with a paper wick soaked with water. Proteins were then focused until 60 KVh-1. Paper wick with remaining protein was kept and re-used to run a 4–7 pH range strip with the same protocol. In a 6–11 pH range, only few proteins were observed (Figure 5C), with a dark background at the bottom of the 2D gel. Nevertheless, this strategy allowed the separation of the abundant milk proteins in the 4–7 pH range (Figure 5B).

To improve protein focalisation following paper-bridge loading, we tried to remove paper wick bonds between strip and electrode during protein focalisation [8]. With this strategy (Figure 5D), the intensity of some spots around 30 kDa decreased slightly, but a lot of new spots appeared after pH9, and a clear background was observed at the bottom of the gel. These new spots were in fact present as well in the 3–10 pH range performed in a first time with protein loading during strip rehydration (Figure 5A), but with such large pH range, spots were less separated and were certainly superposed.

## Conclusion

As attempted, basic protein focalization with IPG strips was a challenge, giving contrasted and surprising results as a function of the protocol strategy used. After the exclusion of the in-gel loading protocol which is definitively inappropriate for basic pH range, the cup-loading protocol clearly displayed the best resolution and reproducibility when the proteins were rehydrated with destreak reagent, and the focalisation was performed with cathodic wicks filled with rehydration solution coupled with DTT. Paper-bridge loading could be as well used, but preferentially with non-limited samples owing to the loss of protein during active rehydration of IPG strips.

## Methods

### Chemicals

TRIS base, urea, thiourea, CHAPS, iodoacetamide, TEMED, low-melt agarose, Triton X-100, spermine, phosphatase inhibitor cocktail and bromophenol blue were obtained from Sigma-Aldrich (St. Louis, MO, USA); protease inhibitor cocktail (Complete Mini EDTA-free) was from Roche Diagnostics (Mannheim, Germany); IPG buffers, IPG strips (pH 6–9 and 6–11) and destreak reagent were purchased from GE (GE Healthcare); acrylamide was obtained from Bio-Rad (Hercules, CA); SDS, glycerol, DTT and TGS 10X were from Eudomedex (Mundolshein, France); all other reagents were of analytical grade.

### Protein extraction and solubilisation

Proteins were extracted from MCF7 cells as previously described [18] in sample buffer containing 7 M urea, 2 M thiourea, 4% CHAPS, 0.05% Triton X100, 65 mM DTT, 40 mM spermine, protease and phosphatase inhibitor cocktails. This suspension was centrifuged at 68000 rpm for 60 min, supernatants were collected and the protein content was estimated using the Bradford method [19].

Proteins were then precipitated using the 2D clean-up kit (GE Healthcare) and the pellet was solubilized with TUC solution (7 M urea, 2 M thiourea, 4% CHAPS) and quantified with the 2D quant kit (GE Healthcare).

### Strip rehydration with protein samples: “sample in-gel rehydration”

Protein sample (50 μg) was mixed with rehydration buffer (RB): 7 M urea, 2 M thiourea, 4% CHAPS, 0.05% triton X100, 5% glycerol complemented with 1.2% destreak reagent, 0.5% ampholytes (IPG buffer 6–11 GE) and adjusted to the correct volume to rehydrate 18 cm strip (here, 320 μl). Strips were then placed acrylamide face down (Figure 6A) in the focusing tray equipped with platinum electrode embedded into the running tray (Protean IEF, Bio-Rad) and passively rehydrated at 20°C without electricity for 16 hours, and then actively rehydrated at 50 V during 9 hours. During protein focalisation, small electrode wicks were placed between acrylamide and electrode. These paper wicks (Ref 1654071, Electrode wicks, Bio-Rad) were in advance soaked with water in order to absorb salts and other contaminant species during active rehydration. The IPG strips were then focused according to the following program: 500 V for 1 h, a linear ramp to 1000 V for 1 h, a linear ramp to 10000 V for 33 KV^-1^ h, and finally 10000 V for 24 KV^-1^ h.

### Cup loading of protein samples

Strips were first rehydrated without proteins using 320 μl of RB with 0.5% IPG buffer 6–11), with 160 mM DTT, or 1.2% destreak reagent and/or 10% iso-propanol at 20° for 16 hours. Following this passive rehydration, strips were removed from the rehydration tray, drained from excess of mineral oil and positioned with the acrylamide face on top, gel side up (Figure 6B) in a “cup loading tray” (Protean IEF, Bio-Rad). Plastic cup were placed at the surface of the acrylamide part, 10 mm away from the anodic end and filled with proteins sample. A paper wick (30 x 4 mm, cut from Blot paper TE70, ref TE76, GE), soaked with 50 μL of water was placed at the anodic extremity and a paper wick soaked with 50 μL of solution (water, rehydration buffer with or without 160 mM DTT) to create a bridge between the acrylamide of strip and the movable electrode (Bio-Rad). The IPG strips were then focused according to the following program: active rehydration at 50 V for 9 hours, then, 500 V for 1 h, a linear ramp to 1000 V for 1 h, a linear ramp to 10000 V for 33 KV^-1^ h, and finally 10000 V for 24 KV^-1^ h.

### Paper-bridge loading of protein samples

Another possibility consisted to use the “cup loading protocol” described earlier, but instead of using a cup to load proteins, proteins were loaded using the anodic paper wick as described by Kane et al. [8], with some adaptations. In this case, strips were first rehydrated using 320 μl of RB with 1.2% destreak reagent and 0.5% IPG buffer 6–11 at 20° for 16 hours. Following this passive rehydration, strips were removed from the rehydration tray, drained from excess of mineral oil and positioned with the acrylamide face on top, gel side up (Figure 6B) in a “cup loading tray” (Protean IEF, Bio-Rad). A paper wick (30 x 4 mm, cut from Blot paper TE70, ref TE76, GE), was placed at each extremity to create a bridge between the acrylamide of strip and the movable electrode (Bio-Rad). Protein sample (adjusted to 50 μl with rehydration buffer) was applied onto the anodic paper wick and 50 μL of rehydration buffer with 160 mM DTT was applied onto the cathodic paper wick. The IPG strips were then focused according to the following program: 200 V for 16 hours, 500 V for 1 h, a linear ramp to 1000 V for 1 h, a linear ramp to 10000 V for 33 KV^-1^ h, and finally 10000 V for 24 KV^-1^ h.

### IPG strips equilibration and second dimension

The strips were incubated two times 10 min in the first equilibration solution (50 mM Tris–HCl pH 8.8, 6 M urea, 30% (v/v) glycerol, 2% (w/v) SDS) with 130 mM DTT and then two times 10 min in the second equilibration solution (50 mM Tris–HCl pH 8.8, 6 M urea, 30% (v/v) glycerol, 2% (w/v) SDS) with 130 mM iodoacetamide.

**Figure 7:**
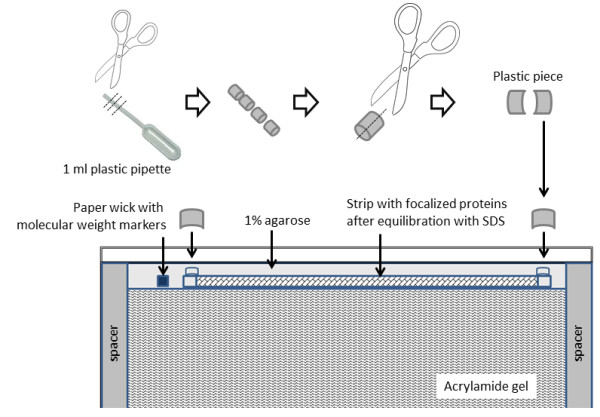
**Schematic of IPG strip placement before the second dimension.** A special precaution is presented to seal strips with 1% agarose and curved plastic pieces.

As described in Figure 7, strips were then embedded using 1% (w/v) low-melt agarose on the top of the acrylamide gel and trapped using plastic blockers. These curved plastic pieces allowed a perfect alignment of the strip with the acrylamide gel, and prevented any displacement before agarose reticulation. A molecular weight marker was prepared with 5 μl of unstained precision plus protein standards (Bio-Rad) on a paper wicks (Ref 1654071, Electrode wicks, Bio-Rad), sealed with a drop of 1% (w/v) low-melt agarose and inserted in the left part of the assembly (Figure 7). SDS-PAGE was carried out on a 12% acrylamide gel (size 20 x 20 cm and 1 mm thickness), using the Dodeca Cell electrophoresis unit (Bio-Rad) at 85 constant voltage, overnight, at 10°C.

### Gel staining and picture acquisition

Gels were stained with silver nitrate as previously described [20,21], with some modifications. Briefly, gels were first fixed at least 1 hour with 30% ethanol and 5% acetic acid; washed 3 times 10 min with water; sensibilized 1 min with 0.02% sodium thiosulfate; washed 2 min with water; stained 30 min with 0.2% silver nitrate and 0.011% formaldehyde; washed 10 s with water; developed 5 min with 85 mM sodium carbonate, 0.00125% sodium thiosulfate and 0.011% formaldehyde; stopped with 0.33 M TRIS and 1.7% acetic acid; stored with 5% acetic acid with 2% DMSO.

Gels were scanned to images right after staining to limit polychromatic colour of spots. Images were acquired with a GS 800 densitometer (Bio-Rad). After several hours in storage solution, gels were dried between sheets of cellophane, using the GelAir drying system (Bio-Rad).

### Image analysis

Images from stained gels were analysed using the Samespots software v4.5 (Non-linear Dynamics, UK). Gel were grouped to create an global analysis with all conditions. Spots of each samples were compared between conditions, and spots were numbered with the same detection parameters.

## Abbreviations

RB: Rehydration buffer; TUC: Thiourea Urea CHAPS.

## Competing interests

The authors declare that they have no competing interests.

## Authors’ contributions

JD and FC performed the experiments. FC designed the experiments and wrote the manuscript. Both authors read and approved the final manuscript.
